# Photoelectrocatalysis on TiO_2_ meshes: different applications in the integrated urban water management

**DOI:** 10.1007/s11356-021-12606-5

**Published:** 2021-02-11

**Authors:** Maria Cristina Collivignarelli, Alessandro Abbà, Marco Carnevale Miino, Giorgio Bertanza, Sabrina Sorlini, Silvestro Damiani, Hamed Arab, Massimiliano Bestetti, Silvia Franz

**Affiliations:** 1 Department of Civil Engineering and Architecture, University of Pavia, Via Ferrata 3, 27100 Pavia, Italy; 2 Interdepartmental Centre for Water Research, University of Pavia, Via Ferrata 3, 27100 Pavia, Italy; 3 Department of Civil, Environmental, Architectural Engineering and Mathematics, University of Brescia, Via Branze 43, 25123 Brescia, Italy; 4 Department of Chemistry, Materials and Chemical Engineering “Giulio Natta”, Politecnico di Milano, Via Mancinelli 7, 20131 Milano, Italy

**Keywords:** PEC, Drinking water, Wastewater, Integrated urban water management, Catalyst, Emerging contaminants, WWTP effluent

## Abstract

Recently, among AOPs, photoelectrocatalysis (PEC) on TiO_2_ is gaining interest. In this study, five different real waters sampled in four different points of the integrated urban water management (IUWM) system were tested with PEC and UV alone, for comparison. This work aims to verify the effect of the PEC suggesting the optimal position in IUWM system where the PEC should be located to obtain the best performance. In groundwaters (GWs), PEC effectively removed atrazine-based compounds (> 99%), trichloroethylene, and perchloroethylene (96%), after 15 min of reaction time. However, given the low concentrations of emerging compounds, the synergistic effect of UV radiation with the catalyst and with the polarization of the mesh was not visible, with very few differences compared with the results obtained with UV alone. Pharmaceutical industrial wastewater (IWW) showed a significant increase in biodegradability after 2 h, both if subjected to PEC or UV (200%), despite the absence of COD removal. The PEC applied on IWW from a sewage sludge treatment plant allowed to effectively remove the COD (39.6%) and increase the biodegradability (300%). Good results in terms of COD removal (33.9%) and biodegradability increase (+900%) were also achieved testing PEC on wastewater treatment plant effluent. Except for GWs, PEC allowed significant *E*_EO_ savings respect to UV alone (76.2–99.1%).

## Introduction

In recent years, the application of advanced oxidation processes (AOPs) on waters is gaining interest (Vilar et al. 2017). In fact, the production of highly reactive OH• proved to effectively remove persistent organic pollutants and recalcitrant substances (Sorlini et al. 2020). Among the AOPs, the application of photoelectrocatalysis (PEC) on waters is still at an early stage of study, but there are several examples of laboratory-scale applications (Wei et al. 2017; Garcia-Segura and Brillas 2017; Fernández-Domene et al. 2018; Brugnera et al. 2019).

PEC is based on the adoption of heterogeneous photocatalysis (PC) combined with the application of a positive bias to the photoanode (Collivignarelli et al. 2020). Thanks to UV irradiation of the catalyst; some electrons occupying the valence band can be excited to the conduction band producing strongly oxidizing holes, necessary for hydroxyl radical production. In PC, the spontaneous electron-hole recombination generally hinders the OH• production. On the contrary, in PEC, the use of an electrical bias allows to reduce this phenomenon increasing the effectiveness of the process (Daghrir et al. 2012; Garcia-Segura and Brillas 2017).

Generally, in conventional photocatalysis (PC), catalyst was used in powdered form with the necessity of a subsequent phase of settling. In PEC, the immobilization of the catalyst allows to overcome this problem (Franz et al. 2015). Supported semi-conductors exhibit a reduced surface area with respect to powdered catalysts, but the enhancement of OH• production due to the reduced electron-hole recombination completely compensates this aspect (Franz et al. 2020a).

In literature, several materials have been tested as photocatalysts in PEC, among them WO_3_, ZnO, MgO, Fe_2_O_3_, and SnO_2_ (Fresno et al. 2014; Fu et al. 2018). However, TiO_2_ represents one of the most studied due to the high catalytic activity, the non-toxicity, good photochemical stability, and the low cost (Komtchou et al. 2016; Franz et al. 2020a). TiO_2_ can be synthetized by different techniques. In this work, plasma electrolytic oxidation (PEO) was chosen due to its advantages such as high growth rates, instantaneous oxide crystallization, and incorporation of chemical species from the electrolyte (Bayati et al. 2010; Franz et al. 2016; Murgolo et al. 2019).

There are several points in the integrated urban water management (IUWM) system where the PEC could potentially be applied. Given the increasingly stringent regulations (Sorlini et al. 2019), some studies have focused on the application of the PEC on DW to remove emerging contaminants with very promising results (Ghasemian et al. 2017; Murgolo et al. 2019; Montenegro-Ayo et al. 2019). In this case, the scope of PEC application is to act on contaminants that are not effectively removed by conventional processes in drinking water treatment plants (DWTPs) (e.g., pesticides and PFAS) or to enhance the removal of microorganism.

In IUWM, industrial wastewaters (IWWs) could represent a problem due to the presence of a wide range of pollutants (Wang and Wang 2016; Burakov et al. 2018; Torres et al. 2019; Collivignarelli et al. 2019b; Ricciardi et al. 2020; Sözen et al. 2020). Conventional treatments (e.g., conventional active sludge (CAS)) are generally unsuitable to remove recalcitrant pollutants (Al-Momani et al. 2002; Quan et al. 2004). Among AOPs, PEC could be a promising alternative thanks to the high efficiency in terms of OH• production. So far, the few studies conducted seem to demonstrate good results in terms of chemical oxygen demand (COD), dyes, and personal care product removal (Franz et al. 2015; Cardoso et al. 2016; Garcia-Segura and Brillas 2017). Moreover, preliminary tests showed a significant increase of the biodegradability of IWWs by a mesophilic biomass after PEC treatment, demonstrating a good complementarity with CAS process in wastewater treatment plants (WWTPs) (Collivignarelli et al. 2020).

Due to promising results of PEC on DW and WW, the application also as a polishing treatment in WWTPs could represent an interesting option to enhance effluent quality promoting the final reuse of water in a circular economy perspective. In fact, the strong oxidizing power of OH• produced by PEC could play a key role in the removal of the residual organic substance and microorganism (Daghrir et al. 2012; Collivignarelli et al. 2021).

Despite the good results present in literature, to date, there is no full-scale PEC system application, but the process is still studied at the laboratory scale. In recent years, several studies focus on application of PEC on polluted water (Garcia-Segura and Brillas 2017; Murgolo et al. 2019; Brugnera et al. 2019; Montenegro-Ayo et al. 2019), but most researches focus on the effect of photoelectrocatalytic treatment only on specific pollutants present in synthetic matrices.

This work aims to verify the effect of the PEC on real waters sampled in different points of the IUWM system, suggesting the optimal position where the PEC should be located to obtain the best performance. Two groundwaters (GWs) were treated for trichloroethylene (TCE), perchloroethylene (PCE), and atrazine-based compounds (ATBC) removal. Moreover, the effect of PEC on two IWWs and a wastewater treatment plant effluent (WWTPE) was tested to remove organic substances and study the effluent biodegradability. Tests with UV alone were also made for comparison. Finally, the specific energy consumptions were compared and discussed.

## Materials and methods

### Water characteristics

**Fig. 1:**
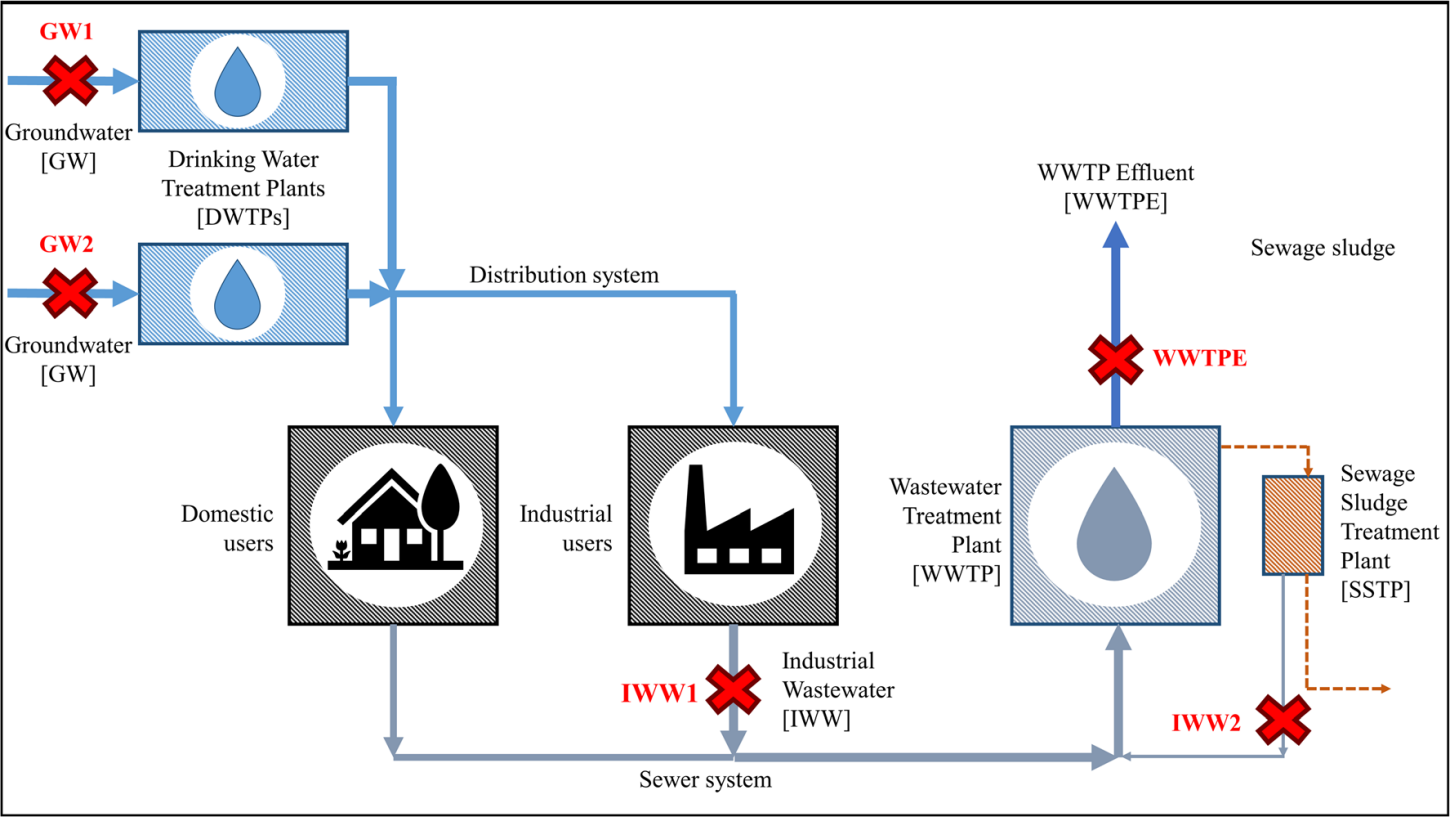
Sampling points of the waters tested in different parts of the IUWM system

In this study, real GWs, IWWs, and WWTPE were used (Table 1). GWs were collected in two different real DW wells before any treatment in drinking water treatment plant (DWTP) and presented contaminations of TCE and PCE (GW1) and ATBC (GW2), respectively. The IWW1 and IWW2 were collected from a company that produces chiral materials for the pharmaceutical industry and from a sewage sludge treatment plant (SSTP), respectively. The WWTPE was the discharge of a WWTP authorized to treat both civil WW and IWW (Fig. 1).

**Table 1:** Chemical and biochemical properties of the waters

	Groundwater		Industrial wastewater		Wastewater treatment plant effluent
Parameter	GW1	GW2	IWW1	IWW2	WWTPE
TCE+PCE (μg L^−1^)	25–30	< 0.5	n.a.	n.a.	n.a.
AT (μg L^−1^)	< 0.01	0.15–0.16	n.a.	n.a.	n.a.
DAT (μg L^−1^)	< 0.01	0.12–0.15	n.a.	n.a.	n.a.
ATD (μg L^−1^)	< 0.01	0.04–0.1	n.a.	n.a.	n.a.
DEAT (μg L^−1^)	< 0.01	0.07–0.1	n.a.	n.a.	n.a.
DACT (μg L^−1^)	< 0.01	0.22–0.30	n.a.	n.a.	n.a.
COD (mg L^−1^)	0.2–0.4	0.1–0.5	1700–2000	1750–1950	123–127
Turbidity (NTU)	< 1.0	< 1.0	< 1.0	< 1.0	< 1.0
pH (−)	7.0–7.5	7.2–7.9	7.8–8.0	7.1–7.5	7.9–8.0
EC^**(b)**^ (mS cm^−1^)	0.6–0.70	0.65–0.7	10.9–11.2	9.5–10.5	0.05–1.33

TCE, trichloroethylene; PCE, perchloroethylene; AT, atrazine; DAT, desethylatrazine; ATD, atrazine-desisopropyl; DEAT, desethyl terbuthylatrazine; DACT, desethyl-desisopropylatrazine; EC, electrical conductivity; n.a., not available

### Laboratory-scale reactor

**Fig. 2:**
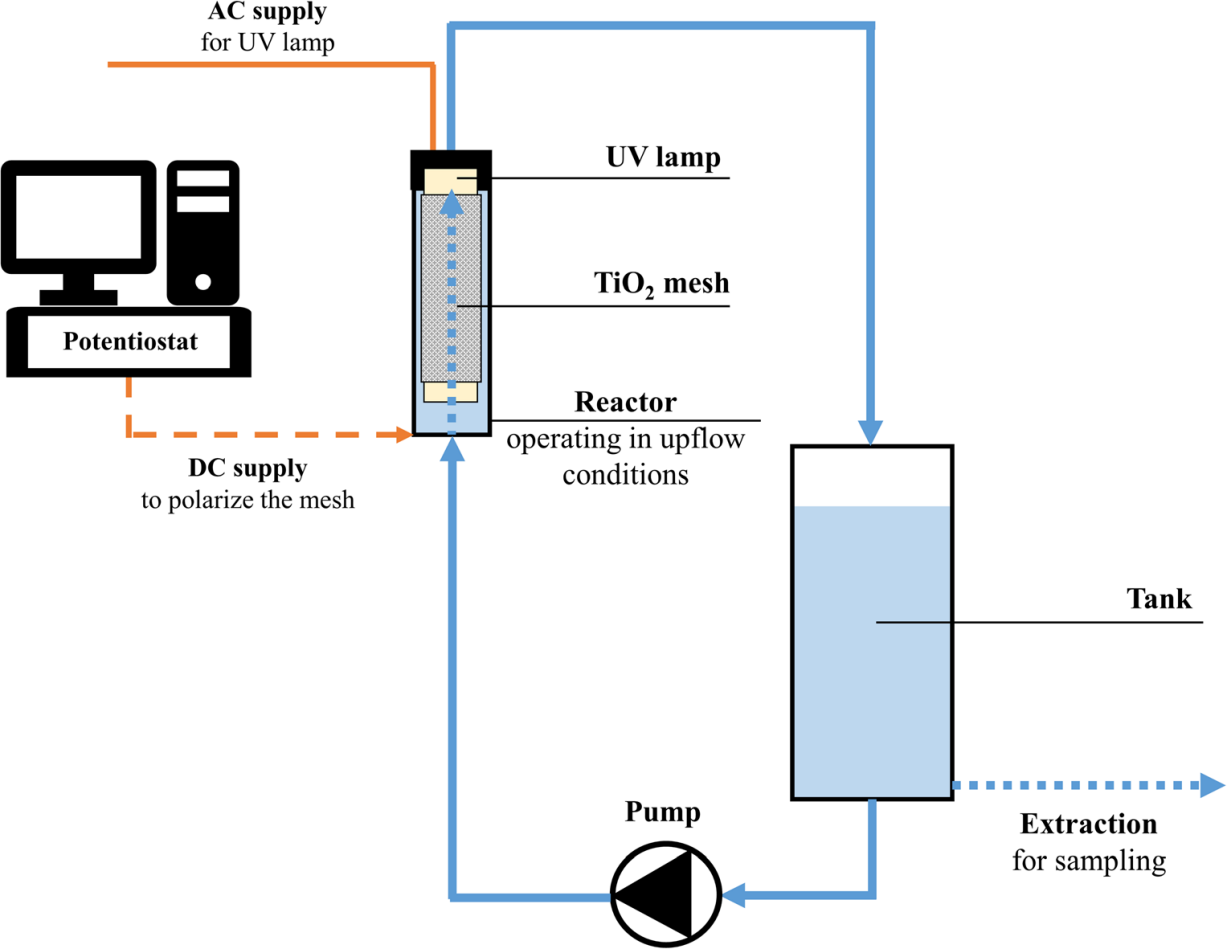
Scheme of the laboratory-scale electrochemical reactor working in up-flow condition and semi-batch mode

Tests were carried out in a laboratory-scale tubular photocatalytic reactor equipped with a buffer reservoir working in semi-batch mode (Fig. 2). An Iwaky Magnet Pump MD-30RZ-220N with a nominal power of 80 W was used to recirculate the water in the system. The reactor was equipped with a 30 W low-pressure Hg vapor UV-C lamp (Helios Italquartz) emitting at 254 nm. Further details on geometrical characteristics of the reactor are available in our previous studies (Murgolo et al. 2019; Franz et al. 2020b; Collivignarelli et al. 2020).

The TiO_2_ mesh was prepared by PEO following Franz et al. (2016) and was kept at a distance of 1 mm from the quartz sheath. The shielding effect of the mesh against UV radiation was about 50% (Murgolo et al. 2019). The electrochemical surface area of the catalyst (ECSA) was 60 cm^2^ cm^−2^. The ECSA per unit mass was about 5.2 m^2^ g^−1^ similar than Brunauer–Emmett–Teller (BET) surface value (6.3 m^2^ g^−1^) (Franz et al. 2020a). Other information about the characterization of TiO_2_ catalyst are available in Murgolo et al. (2019), Franz et al. (2020a), and Collivignarelli et al. (2020) During the PEC tests, the anodized titanium mesh was anodically polarized while the reactor body was cathodically polarized, and a constant cell voltage of 4 V was applied by means of a potentiostat/galvanostat (AMEL 2549). The same instrument allowed continuous chronoamperometric monitoring of the photoelectrochemical activity of the TiO_2_ mesh.

### Analytical methods

TCE and PCE in GW1 were determined by means of gas chromatography coupled with static and dynamic headspace. Instead, ATBC were determined in gas chromatography with NPD detector, according to Italian standards (APAT-IRSA/CNR 2003). Atrazine (AT), desethylatrazine (DAT), atrazine-desisopropyl (ATD), desethyl terbuthylatrazine (DEAT), and desethyl-desisopropylatrazine (DACT) in GW2 were monitored by HPLC-DAD (Waters 2695) using a WatersTM Spherisorb 5-mm ODS2 column (4.6-mm i.d. and 250-mm length). ATBC was calculated as the sum of AT, DAT, ATD, DEAT, and DACT.

COD in IWW and WWTPE was measured according to the *Standard Methods for the Examination of Water and Wastewater* (APHA 2012). For the analysis, kits purchased from Hach Company were used. Before the COD analysis, the interference from chlorides was ruled out by verifying that the chloride concentration was lower than the maximum accepted by the method. pH and electrical conductivity (EC) of the untreated waters were measured using a portable multiparameter instrument (WTW 3410 SET4). pH was measured using the probe WTW-IDS, Model SenTix® 940, and EC was measured using the probe WTW-IDS, model TetraCon® 925.

The specific oxygen uptake rate (SOUR) tests were carried out at 20 °C adopting the ISO 8192 procedure (ISO 8192 2007), using a mesophilic biomass taken from a WWTP (authorized to treat both municipal and industrial WW), the same in which the WWTPE was collected.

### Experimental procedure and data processing

Two different working conditions were tested:

(A) UV: photolysis (PL)

(B) UV/TiO_2_/bias 4V: photoelectrocatalysis (PEC)

In order to eliminate the volatilization phenomena, the water supply tank was hermetically sealed by means of a polymeric cap. The tests on GWs lasted 15 min while tests on IWWs and WWTPE were carried out for 2 h of reaction time. During the treatment, several samples were collected using glass vials. 

The processes efficiency was calculated as (Eq. 1):

1 $$ Removal\ yield\ \left[\%\right]=\frac{L_0-{L}_{\mathrm{i}}}{L_0}\ast 100 $$

where *L*_0_ and *L*_i_ represent the initial and the currently i-th load of pollutant, respectively.

Considering that the SOUR value is a function of the quantity of organic substance present in the tested water (Zheng et al. 2016; Inglezakis et al. 2017), a specific biodegradation rate (SBR) was calculated relating the SOUR value (mgO_2_ g_VSS_^-1^ h^-1^) to the COD (g_COD_) tested, in order to compare different matrices (Eq. 2):

2 $$ SBR\ \left[m{g}_{{\mathrm{O}}_2}\ {g}_{\mathrm{COD}}^{-1}\ {g}_{\mathrm{VSS}}^{-1}\ {h}^{-1}\right]=\frac{SOUR\ }{COD\ } $$

Moreover, for all tested waters, the energy consumption was correlated with the pollutants removal as electrical energy per order (*E*_EO_) in (kW m^−3^ order^−1^), according to Eq. 3 (Bolton et al. 2001; Bessegato et al. 2018):

3 $$ {E}_{\mathrm{EO}}\left[ kW\ h\ {m}^{-3}\ {order}^{-1}\right]=\frac{P\ast t\ast {10}^3}{V\ast {\log}_{10}\left(\frac{C_i}{C_f}\right)} $$

where *P* is the nominal power (kW) of the system, *t* (h) is the processing time, *V* (L) is the volume of water treated, and *C* represents the concentration of COD. The nominal power (*P*) was assumed equal to the energy consumption of the UV lamp. In GW1 and GW2, the energy consumption was correlated with TCE+PCE and ATBC removal, respectively. Instead, in IWW and WWTPE, the energy consumption was related with COD removal using the same equation (Eq. 3).

For comparative purposes, the *E*_EO_ saved using PEC despite the UV alone was calculated as reported in the following equation (Eq. 4):

4 $$ {E}_{\mathrm{EO}}\ \mathrm{saved}\ \left[\%\right]=\left(1-\frac{E_{\mathrm{EO}\ \mathrm{PEC}}}{E_{\mathrm{EO}\ \mathrm{UV}}}\right)\ast 100 $$

## Results and discussion

### Application on groundwater

**Fig. 3:**
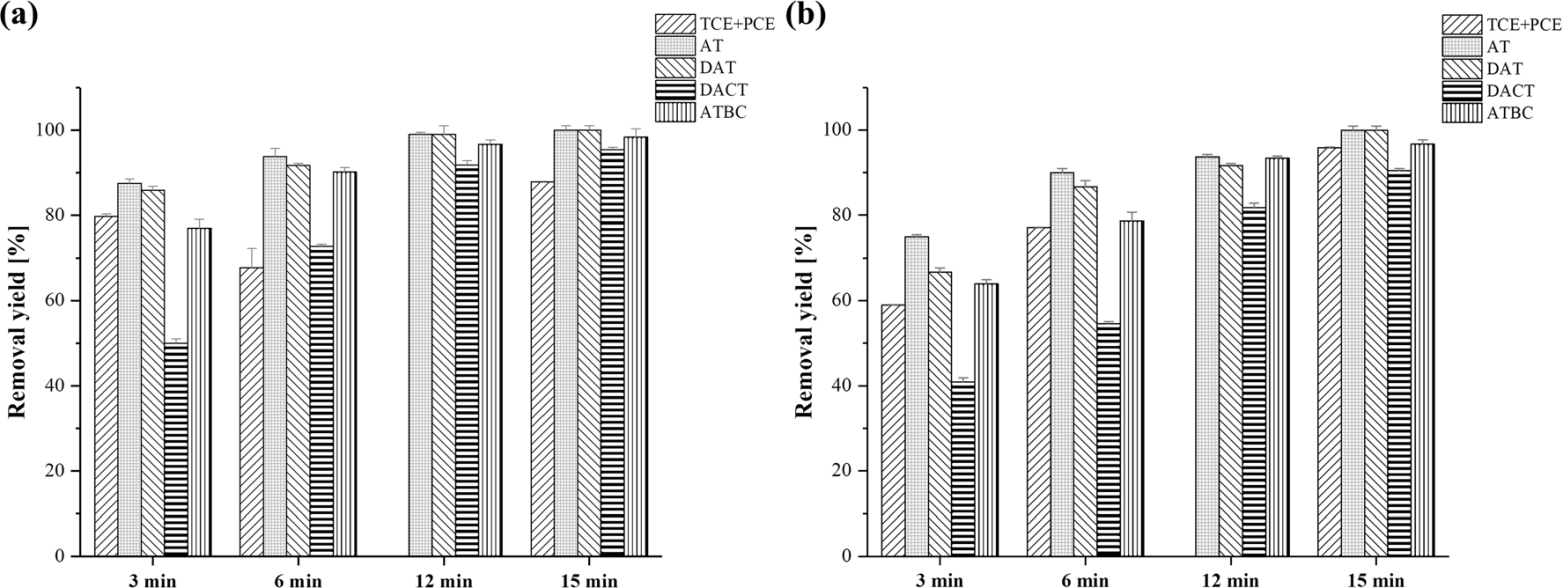
TCE+PCE, AT, DAT, DACT, and ATBC removal yields after **a** UV process or **b** PEC process. The red bars represent the 95% confidence interval. AT, atrazine; ATBCs, atrazine-based compounds; DACT, desethyl-desisopropylatrazine; DAT, desethylatrazine; PCE, perchloroethylene; TCE, trichloroethylene

Two real GWs were treated with PEC in order to study the effectiveness of the process on TCE+PCE and atrazine-based herbicide compound (as sum of AT, DAT, ATD, DEAT, and DACT) removal. The results were compared with those obtained with UV process (Fig. 3).

Regarding the removal of TCE and PCE, faster kinetics were observed in PEC, however, without great difference compared with photolysis alone. In fact, the removal yields of TCE+PCE were 88% and 96% after just 15 min of reaction time with UV and PEC, respectively. The literature confirms these results reporting that photolytic process is effective on the removal of TCE and PCE (Rashid and Sato 2011), and the addition of the TiO_2_ catalyst in immobilized form increases the efficiency of the process (Grzechulska-Damszel et al. 2014; Franz et al. 2020b).

Atrazine-based herbicide compounds were effectively removed by UV and PEC with similar results (Fig. 3). AT and DAT were completely removed by both processes after 12–15 min of reaction time. At the same time, UV showed a slightly better removal yield of DACT (95%) with respect to the PEC (90%). Due to the low initial concentration, ATD and DEAT were measured during the tests, but their removal yields are not shown. Considering also these two compounds, after 15 min of reaction time, both PEC and UV showed the total removal of ATBC. No significant differences in term of kinetics using photolysis or PEC for ATBC removal were highlighted.

The main reason for this result could be due to the initial concentrations of ATBC. Given the low concentrations in which the atrazine-based herbicide compounds found in GW2 (in the order of μg L^−1^, typical of a real GW (Almberg et al. 2018)), the synergistic effect of UV radiation with the catalyst and with the polarization of the mesh was not visible. Therefore, the use of PEC on DW is recommended only with a higher concentration of ATBC (e.g., in surface water). As a comparison, the literature confirms the possible removal of atrazine both by photolysis and by PEC with TiO_2_ (Moreira et al. 2017; Komtchou et al. 2018; Wang et al. 2019).

### Application on industrial wastewater and WWTP effluent

**Fig. 4:**
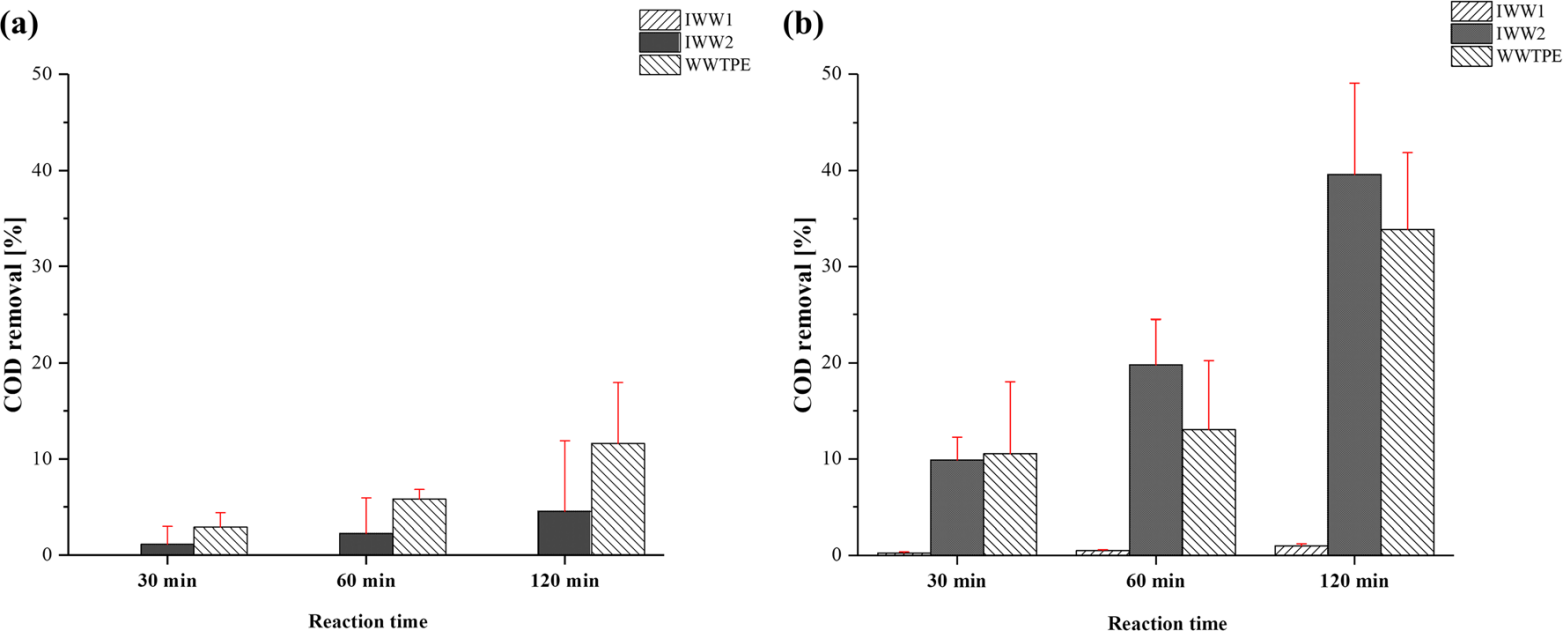
COD removal yields after **a** UV process or **b** PEC process. The red bars represent the 95% confidence interval

The PEC has been tested on two IWWs and a WWTPE. After 2 h of reaction time, both PEC and UV did not produce COD removal on the pharmaceutical WW (Fig. 4). This result was confirmed by the literature that classified these waters as particularly recalcitrant and treatable effectively with PEC and UV but only with much longer contact times unless adding other oxidants such as hydrogen peroxide (Collivignarelli et al. 2020). On the contrary, with IWW_2_ and WWTPE, the PEC showed a greater ability to remove the COD compared with UV alone. In fact, 39.6% and 33.9% of the COD IWW2 and WWTPE, respectively, were removed (Fig. 4).

**Fig. 5:**
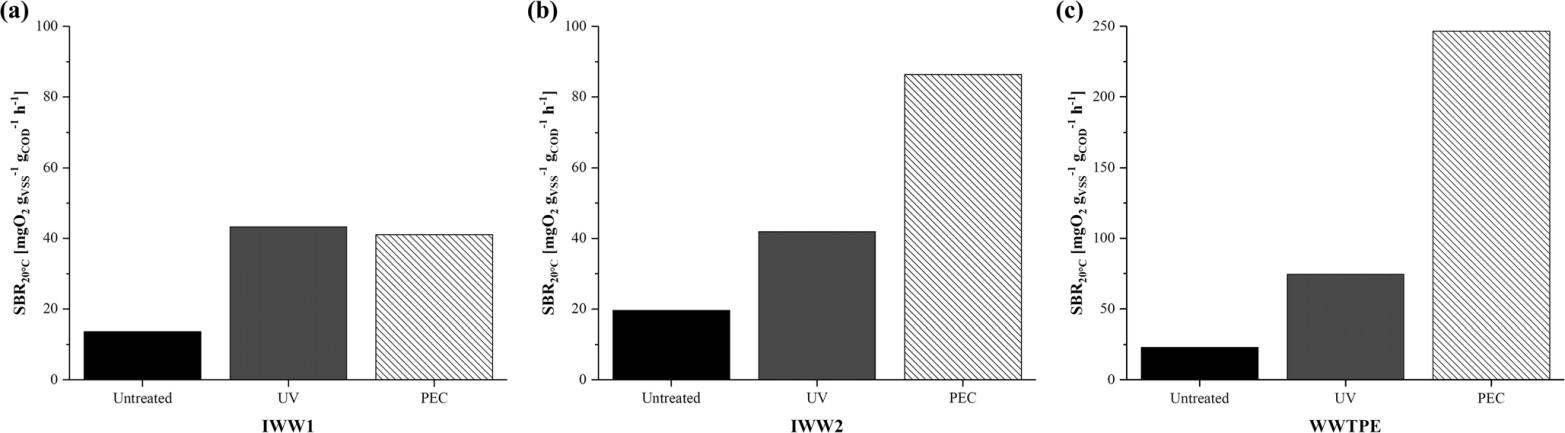
Specific biodegradation rate (SBR) for **a** IWW1, **b** IWW2, and **c** WWTPE of the untreated waters and after 2 h treatment with UV or PEC

Considering that the SOUR value which indicates the biodegradability of a matrix by a biomass is a function of the quantity of organic substance present in the tested water (Zheng et al. 2016; Inglezakis et al. 2017), a specific rate has been calculated relating the SOUR value to the COD (please, refer to “Materials and methods” for further details on SBR calculation). Regarding IWW1, both processes were able to increase the biodegradability of the wastewater by tripling the SBR value despite the absence of COD removal (Fig. 5). This result can be related with the ability of the photolysis and PEC processes to split long-chain organic molecules and make them more degradable by biomass. However, despite a significant increase in SBR value with respect to untreated matrix was observed, the PEC process on pharmaceutical IWW after 120 min of reaction time was not able to guarantee better performance than the photolysis process alone.

On the contrary, in IWW2 and WWTPE, a higher increase of SBR (Fig. 5) can be highlighted after PEC (+300% and +900%, respectively) rather compared with that obtained with UV alone (+100% and +200%, respectively). In the case of IWW2, a good biodegradability of the organic substance means a better acceptability of the WW from a hypothetical CAS system present in a WWTP and therefore a better treatment capacity. This result was due to the higher oxidizing redox potential of OH^●^ hydroxyl radicals (2.60 V), produced during the PEC, with respect to the photolytic activity of UV rays alone (Eqs. 5, 6, and 7) (Kistan et al. 2018; Collivignarelli et al. 2019a):

5 $$ Ti{O}_2+ UV\to {e}_{cb}^{-}+{h}_{vb}^{+}\to \varDelta T $$

6 $$ {h}_{vb}^{+}+O{H}^{-}\to O{H}^{\bullet } $$

7 $$ {h}_{vb}^{+}+{H}_2O\to O{H}^{\bullet }+{H}^{+} $$

This aspect was even more evident in the case of WWTPE treated with PEC on TiO_2_ mesh and has a significant importance. In fact, the increase in the biodegradability of the effluent involves a lower impact on the ecosystem as the organic substance contained in the effluent becomes more easily biologically removable in mesophilic conditions by microorganisms naturally present in water.

### Energy consumption

In all PEC tests, the impact of the bias on the total energy consumption can be considered negligible (around 2% of the total energy required) due to the low electric current (200–300 mA) and the low voltage (4 V) required. This result was confirmed by our previous studies. In fact, Collivignarelli et al. (2020) found that the impact of the bias was almost the 0.3% of the total energy required. For this reason, in this work, the energy consumption due to the usage of bias was neglected.

The values of *E*_EO_ after 2 h of treatment and the *E*_EO_ saved using PEC with respect to photolysis are reported in Table 2. Based on the results, it can generally be concluded that the photoelectrocatalytic reaction was more efficient in terms of energy consumption than photolysis for all waters excluding GW2. By applying a photoelectrocatalytic process on GW containing TCE and PCE with respect to photolysis, the *E*_EO_ saved was equals to 34.1%. Instead, when GW containing ATBC was treated by PEC, the *E*_EO_ required was 20% higher if compared with photolysis. These results were due to the absence of the synergistic effect of UV radiation with the catalyst and with the polarization of the mesh. The application of the PEC on IWW1, IWW2, and WWTPE ensured a very high specific energy consumption savings compared with those necessary for the treatment of these same matrices with UV alone (99.1%, 90.9%, and 76.2%, respectively).

**Table 2:** Values of electrical energy per order (*E*_EO_ kW m^−3^ order^−1^) after 2 h of PEC and ratios between *E*_EO_ required in PEC and correspondent values for UV process

Water tested	Pollutants removed	*E*_EO PEC_ (kW h m^−3^ order^−1^)	*E*_EO_ saved (%)
GW1	TCE + PCE	5.4	34.1
GW2	ATBC	5.1	−20.2
IWW1	COD	296.9	99.1
IWW2	COD	274.1	90.9
WWTPE	COD	297.6	76.2

TCE, trichloroethylene; PCE, perchloroethylene; ATBC, atrazine-based compounds

However, the *E*_EO_ values were higher in the treatment of IWW (274.1–296.9 kW h m^−3^ order^−1^) and WWTPE (297.6 kW h m^−3^ order^−1^) than in GW (5.1–5.4 kW h m^−3^ order^−1^) as longer contact times were required.

## Conclusions and future outlooks

A laboratory-scale plant was used to test the PEC on TiO_2_ meshes on five real waters of different origins (GW, IWW, and WWTPE) sampled in four different points of the IUWM. This work aims to verify the effect of the PEC suggesting the optimal position in IUWM system where the PEC should be located in order to obtain the best performance. The results were compared with those obtained with the use of UV alone. The results highlighted that the PEC effectively removed the ATBC (> 99%), TCE, and PCE (96%) present in the GW after 15 min of reaction time. Pharmaceutical industrial wastewater (IWW1) showed a significant increase in biodegradability after 2 h, both if subjected to PEC or UV (200%), despite the absence of COD removal. The PEC applied on IWW from SSTP allowed to effectively remove the COD (39.6%) and increase the biodegradability (300%). Good results in terms of COD removal (33.9%) and biodegradability increase (+900%) were also achieved testing PEC on WWTPE.

So far, most studies have involved synthetic waters containing only one single organic pollutant. The present study demonstrates the feasibility of using the PEC also on certain types of real IWWs (e.g., from SSTPs) and WWTPE. On the contrary, photoelectrocatalytitc treatment of GWs seems to be no more effective than UV alone due to the low initial concentration of pollutants that makes the synergistic effect of UV radiation with the catalyst and with the polarization of the mesh hardly visible. However, if it seems that with ATBC concentrations on the order of μg L^−1^, the adoption of an electrochemical process is not justified, several studies showed that photolysis alone was not able to completely remove intermediate compounds caused by degradation by ATBC (Moreira et al. 2017; Rózsa et al. 2019). Instead, Komtchou et al. (2016) reported that PEC seems to be able to remove intermediate degradation compounds in a shorter time. Therefore, other studies on ATBC and other emerging pollutants degradation by-products are strongly suggested before application of PEC on real-scale DWTPs.

PEC could be also applied on surface waters, generally characterized by a higher concentration of pollutants. However, since DW from surface water body is generally rich in organic substances, ad hoc studies are suggested to evaluate the effect of the initial concentration of organic substances on the loss of efficiency of the mesh. A possible future outlook could be the study of the possible competition and entrainment effects in the treatment of waters with two or more different organic pollutants in order to optimize the efficiency of the process, and understand the complex reactions of the organic compounds during the PEC.

Except for GWs, PEC allowed significant *E*_EO_ savings respect to UV alone (76.2–99.1%). However, the *E*_EO_ values were higher in the treatment of IWW (269.9–274.1 kW h m^−3^ order^−1^) and WWTPE (297.6 kW h m^−3^ order^−1^) than in GW (5.1–5.4 kW h m^−3^ order^−1^) as longer contact times were required. The efficiency of the process from an energy point of view is an aspect that requires further studies in view of a hypothetical full-scale application. Recently, the use of solar energy has been proposed as an alternative to UV lamps in PEC technology (Kushwaha et al. 2016; Orimolade et al. 2019; Zhou et al. 2020; Adak et al. 2020). Although this solution would require longer irradiation times (and therefore a greater volume of reactors), it would considerably limit the costs of water treatment thanks to the exploitation of the energy naturally present. To date, studies are in progress to overcome two main problems of this solution: (i) difficulty in obtaining catalysts with a band gap compatible with the spectrum of solar radiation and (ii) the need for large surfaces to allow a sufficiently low water head such as to allow sunlight to penetrate uniformly.

## Data Availability

All data generated or analyzed during this study are included in this published article.

## Abbreviations

AT: atrazine
ATBC: atrazine-based compounds
ATD: atrazine-desisopropyl
BET: Brunauer–Emmett–Teller
COD: chemical oxygen demand
DACT: desethyl-desisopropylatrazine
DAT: desethylatrazine
DEAT: desethyl terbuthylatrazine
DW: drinking water
DWTP: drinking water treatment plant
EC: electrical conductivity
ECSA: electrochemical surface area
*E*_EO_: electrical energy per order
GW: groundwater
IUWM: integrated urban water management
IWW: industrial wastewater
PCE: perchloroethylene
PEC: photoelectrocatalysis
SBR: specific biodegradation rate
SOUR: specific oxygen uptake rate
SSTP: sewage sludge treatment plant
TCE: trichloroethylene
TiO_2_: titanium dioxide
UV: photolysis
WW: wastewater
WWTP: wastewater treatment plant
WWTPE: wastewater treatment plant effluent

## References

[CR1] Adak D, Chakrabarty P, Majumdar P, Mukherjee R, Patra S, Mondal A, Bhattacharyya S, Saha H, Bhattacharyya R (2020). Pd Nanoparticle-decorated hydrogen plasma-treated TiO2 for photoelectrocatalysis-based solar energy devices. ACS Appl Electron Mater.

[CR2] Almberg K, Turyk M, Jones R, Rankin K, Freels S, Stayner L (2018). Atrazine contamination of drinking water and adverse birth outcomes in community water systems with elevated atrazine in Ohio, 2006–2008. Int J Environ Res Public Health.

[CR3] Al-Momani F, Touraud E, Degorce-Dumas J (2002). Biodegradability enhancement of textile dyes and textile wastewater by VUV photolysis. J Photochem Photobiol A Chem.

[CR4] APAT-IRSA/CNR (2003) Analytical methods for water quality control

[CR5] APHA (2012) Standard methods for the examination of water and wastewater, 22nd Editi

[CR6] Bayati MR, Moshfegh AZ, Golestani-Fard F (2010). In situ growth of vanadia–titania nano/micro-porous layers with enhanced photocatalytic performance by micro-arc oxidation. Electrochim Acta.

[CR7] Bessegato GG, de Souza JC, Cardoso JC, Zanoni MVB (2018). Assessment of several advanced oxidation processes applied in the treatment of environmental concern constituents from a real hair dye wastewater. J Environ Chem Eng.

[CR8] Bolton JR, Bircher KG, Tumas W, Tolman CA (2001). Figures-of-merit for the technical development and application of advanced oxidation technologies for both electric- and solar-driven systems (IUPAC Technical Report). Pure Appl Chem.

[CR9] Brugnera MF, Miyata M, Zocolo GJ, Gonçalves Tessaro LL, Fujimura Leite CQ, Boldrin Zanoni MV (2019). A promising technology based on photoelectrocatalysis against Mycobacterium tuberculosis in water disinfection. Environ Technol.

[CR10] Burakov AE, Galunin EV, Burakova IV, Kucherova AE, Agarwal S, Tkachev AG, Gupta VK (2018). Adsorption of heavy metals on conventional and nanostructured materials for wastewater treatment purposes: a review. Ecotoxicol Environ Saf.

[CR11] Cardoso JC, Bessegato GG, Boldrin Zanoni MV (2016). Efficiency comparison of ozonation, photolysis, photocatalysis and photoelectrocatalysis methods in real textile wastewater decolorization. Water Res.

[CR12] Collivignarelli MC, Abbà A, Carnevale Miino M, Damiani S (2019). Treatments for color removal from wastewater: state of the art. J Environ Manage.

[CR13] Collivignarelli MC, Carnevale Miino M, Baldi M, Manzi S, Abbà A, Bertanza G (2019). Removal of non-ionic and anionic surfactants from real laundry wastewater by means of a full-scale treatment system. Process Saf Environ Prot.

[CR14] Collivignarelli MC, Abbà A, Carnevale Miino M et al (2020) Decolorization and biodegradability of a real pharmaceutical wastewater treated by H2O2-assisted photoelectrocatalysis on TiO2 meshes. J Hazard Mater:121668. 10.1016/j.jhazmat.2019.121668 10.1016/j.jhazmat.2019.121668 31784132

[CR15] Collivignarelli MC, Abbà A, Carnevale Miino M (2021). Disinfection of wastewater by UV-based treatment for reuse in a circular economy perspective. Where are we at?. Int J Environ Res Public Health.

[CR16] Daghrir R, Drogui P, Robert D (2012). Photoelectrocatalytic technologies for environmental applications. J Photochem Photobiol A Chem.

[CR17] Fernández-Domene RM, Sánchez-Tovar R, Lucas-granados B, Muñoz-Portero MJ, García-Antón J (2018). Elimination of pesticide atrazine by photoelectrocatalysis using a photoanode based on WO3 nanosheets. Chem Eng J.

[CR18] Franz S, Perego D, Marchese O, Bestetti M (2015). Photoelectrochemical advanced oxidation processes on nanostructured TiO2 catalysts: decolorization of a textile azo-dye. J Water Chem Technol.

[CR19] Franz S, Perego D, Marchese O, Lucotti A, Bestetti M (2016). Photoactive TiO 2 coatings obtained by plasma electrolytic oxidation in refrigerated electrolytes. Appl Surf Sci.

[CR20] Franz S, Arab H, Chiarello GL, Bestetti M, Selli E (2020). Single-step preparation of large area TiO 2 photoelectrodes for water splitting. Adv Energy Mater.

[CR21] Franz S, Falletta E, Arab H, Murgolo S, Bestetti M, Mascolo G (2020). Degradation of carbamazepine by photo(electro)catalysis on nanostructured TiO2 meshes: transformation products and reaction pathways. Catalysts.

[CR22] Fresno F, Portela R, Suárez S, Coronado JM (2014). Photocatalytic materials: recent achievements and near future trends. J Mater Chem A..

[CR23] Fu C-F, Wu X, Yang J (2018). Material design for photocatalytic water splitting from a theoretical perspective. Adv Mater.

[CR24] Garcia-Segura S, Brillas E (2017). Applied photoelectrocatalysis on the degradation of organic pollutants in wastewaters. J Photochem Photobiol C Photochem Rev.

[CR25] Ghasemian S, Nasuhoglu D, Omanovic S, Yargeau V (2017). Photoelectrocatalytic degradation of pharmaceutical carbamazepine using Sb-doped Sn 80% -W 20% -oxide electrodes. Sep Purif Technol.

[CR26] Grzechulska-Damszel J, Grześkowiak M, Przepiórski J, Morawski AW (2014). Photocatalytic decomposition of low-concentrated trichloroethylene and tetrachloroethylene in water. Int J Environ Res.

[CR27] Inglezakis VJ, Malamis S, Omirkhan A, Nauruzbayeva J, Makhtayeva Z, Seidakhmetov T, Kudarova A (2017). Investigating the inhibitory effect of cyanide, phenol and 4-nitrophenol on the activated sludge process employed for the treatment of petroleum wastewater. J Environ Manage.

[CR28] ISO 8192 (2007) Water Quality - Test for inhibition of oxygen consumption by activated sludge for carbonaceous and ammonium oxidation.

[CR29] Kistan A, Kanchana V, Sakayasheela L (2018). Titanium dioxide as a catalyst for photodegradation of various concentrations of methyl orange and methyl red dyes using Hg vapour lamp with constant pH. Orient J Chem.

[CR30] Komtchou S, Dirany A, Drogui P, Delegan N, el Khakani MA, Robert D, Lafrance P (2016). Degradation of atrazine in aqueous solution with electrophotocatalytic process using TiO 2−x photoanode. Chemosphere.

[CR31] Komtchou S, Delegan N, Dirany A, Drogui P, Robert D, el Khakani MA (2018). Removal of atrazine by photoelectrocatalytic process under sunlight using WN-codoped TiO2 photoanode. J Appl Electrochem.

[CR32] Kushwaha HS, Madhar NA, Ilahi B, Thomas P, Halder A, Vaish R (2016). Efficient solar energy conversion using CaCu3Ti4O12 photoanode for photocatalysis and photoelectrocatalysis. Sci Rep.

[CR33] Montenegro-Ayo R, Morales-Gomero JC, Alarcon H, Cotillas S, Westerhoff P, Garcia-Segura S (2019). Scaling up photoelectrocatalytic reactors: a TiO2 nanotube-coated disc compound reactor effectively degrades acetaminophen. Water.

[CR34] Moreira AJ, Borges AC, Gouvea LFC, MacLeod TCO, Freschi GPG (2017). The process of atrazine degradation, its mechanism, and the formation of metabolites using UV and UV/MW photolysis. J Photochem Photobiol A Chem.

[CR35] Murgolo S, Franz S, Arab H et al (2019) Degradation of emerging organic pollutants in wastewater effluents by electrochemical photocatalysis on nanostructured TiO2 meshes. Water Res:114920. 10.1016/j.watres.2019.114920 10.1016/j.watres.2019.114920 31401328

[CR36] Orimolade BO, Koiki BA, Zwane BN, Peleyeju GM, Mabuba N, Arotiba OA (2019). Interrogating solar photoelectrocatalysis on an exfoliated graphite–BiVO4/ZnO composite electrode towards water treatment. RSC Adv.

[CR37] Quan X, Shi H, Liu H, Wang J, Qian Y (2004). Removal of 2,4-dichlorophenol in a conventional activated sludge system through bioaugmentation. Process Biochem.

[CR38] Rashid MM, Sato C (2011). Photolysis, sonolysis, and photosonolysis of trichloroethane (TCA), trichloroethylene (TCE), and tetrachloroethylene (PCE) without catalyst. Water, Air, Soil Pollut.

[CR39] Ricciardi P, Cillari G, Carnevale Miino M, Collivignarelli MC (2020). Valorization of agro-industry residues in building and environmental sector: a review. Waste Manag Res.

[CR40] Rózsa G, Fazekas Á, Náfrádi M, Alapi T, Schrantz K, Takács E, Wojnárovits L, Fath A, Oppenländer T (2019). Transformation of atrazine by photolysis and radiolysis: kinetic parameters, intermediates and economic consideration. Environ Sci Pollut Res.

[CR41] Sorlini S, Collivignarelli MC, Carnevale Miino M (2019). Technologies for the control of emerging contaminants in drinking water treatment plants. Environ Eng Manag J.

[CR42] Sorlini S, Collivignarelli C, Carnevale Miino M, Caccamo FM, Collivignarelli MC (2020). Kinetics of microcystin-LR removal in a real lake water by UV/H2O2 treatment and analysis of specific energy consumption. Toxins (Basel).

[CR43] Sözen S, Olmez-Hanci T, Hooshmand M, Orhon D (2020). Fenton oxidation for effective removal of color and organic matter from denim cotton wastewater without biological treatment. Environ Chem Lett.

[CR44] Torres K, Álvarez-Hornos FJ, Ferrero P, Gabaldón C, Marzal P (2019). Intermittent operation of UASB reactors treating wastewater polluted with organic solvents: process performance and microbial community evaluation. Environ Sci Water Res Technol.

[CR45] Vilar VJP, Amorim CC, Brillas E, Puma GL, Malato S, Dionysiou DD (2017). AOPs: recent advances to overcome barriers in the treatment of water, wastewater and air. Environ Sci Pollut Res.

[CR46] Wang J, Wang S (2016). Removal of pharmaceuticals and personal care products (PPCPs) from wastewater: a review. J Environ Manage.

[CR47] Wang H, Zhu L, Guo F (2019). Photoelectrocatalytic degradation of atrazine by boron-fluorine co-doped TiO2 nanotube arrays. Environ Sci Pollut Res.

[CR48] Wei Z, Liang F, Liu Y, Luo W, Wang J, Yao W, Zhu Y (2017). Photoelectrocatalytic degradation of phenol-containing wastewater by TiO2/g-C3N4 hybrid heterostructure thin film. Appl Catal B Environ.

[CR49] Zheng D, Chang Q, Li Z, Gao M, She Z, Wang X, Guo L, Zhao Y, Jin C, Gao F (2016). Performance and microbial community of a sequencing batch biofilm reactor treating synthetic mariculture wastewater under long-term exposure to norfloxacin. Bioresour Technol.

[CR50] Zhou G, Zhao T, Wang O, Xia X, Pan JH (2020). Bi2Se3, Bi2Te3 quantum dots-sensitized rutile TiO2 nanorod arrays for enhanced solar photoelectrocatalysis in azo dye degradation. J Phys Energy.

